# From 1D microbiological assays to 3D advanced skin models: enhancing preclinical strategies to unravel the impact of bioactive textiles on the human skin microbiome

**DOI:** 10.3389/fcimb.2025.1676663

**Published:** 2025-10-23

**Authors:** Irina Negut, Camilla Mazzanti, Rossella Laurano, Gianluca Ciardelli, Simona Bronco, Cláudia S. Oliveira

**Affiliations:** ^1^ Lasers Department, National Institute for Lasers, Plasma and Radiation Physics, Măgurele, Romania; ^2^ Institute for Chemical-Physical Processes, National Research Council (CNR-IPCF), Pisa, Italy; ^3^ Department of Mechanical and Aerospace Engineering, Politecnico di Torino, Turin, Italy; ^4^ Universidade Católica Portuguesa, CBQF - Centro de Biotecnologia e Química Fina – Laboratório Associado, Escola Superior de Biotecnologia, Porto, Portugal

**Keywords:** bioactive textiles, skin microbiome, 3D skin models, antimicrobial strategies, microbiome-safe materials

## Abstract

Bioactive textiles have emerged as multifunctional materials to actively interact with the human skin and its microbiome. By embedding natural or synthetic bioactive compounds, such as chitosan, essential oils, plant extracts, and metallic nanoparticles, these materials aim to prevent and target infections, modulate inflammation, and promote skin homeostasis. Given the critical role of the skin microbiome in maintaining barrier integrity and immune balance, strategies that selectively inhibit pathogenic microorganisms (e.g., *Staphylococcus aureus*, *Cutibacterium acnes*) while preserving beneficial commensals like *Staphylococcus epidermidis* are essential to avoid dysbiosis and associated dermatological disorders. This review highlights current trends in the design and functionalization of bioactive textiles, emphasizing sustainable and biocompatible approaches that leverage natural antimicrobial compounds and green synthesis techniques. It also examines conventional evaluation pipelines primarily based on 1D microbiological assays and 2D skin models, highlighting their limitations in predicting real-world performance. Advanced *in vitro* models, particularly 3D reconstructed human skin platforms incorporating both pathogenic and commensal microbiota members, are presented as indispensable tools to study fabric–skin–microbe interactions under physiologically relevant conditions. These models enable accurate assessment of antimicrobial efficacy, biocompatibility, and microbiome impact, providing a bridge between *in vitro* and clinical outcomes. Furthermore, the potential of bioactive textiles in managing microbiome-related skin conditions, such as atopic dermatitis and acne, is discussed alongside the importance of developing microbiome-safe materials. Despite encouraging clinical evidence demonstrating pathogen reduction and symptomatic improvement, the successful translation of these materials to clinical practice needs interdisciplinary research and the adoption of advanced preclinical strategies to ensure innovative solutions for personalized skin health.

## 1 Introduction

The human skin microbiome is a critical determinant of skin health, acting as a dynamic interface between the body and the external environment. This complex ecosystem of bacteria, fungi, and viruses is essential for maintaining barrier function, modulating immune responses, and resisting pathogen colonization. Disruptions in this microbial equilibrium, often termed dysbiosis, are implicated in a wide range of dermatological disorders including acne, atopic dermatitis (AD), psoriasis, and chronic wounds (Byrd et al., 2018; Carmona-Cruz et al., 2022). In this context, bioactive textiles have emerged as a promising therapeutic approach for restoring microbial balance while simultaneously addressing rising concerns about antibiotic resistance and environmental sustainability (Suellen Ferro de Oliveira and Kekhasharu Tavaria, 2023).

Bioactive textiles are engineered by incorporating functional agents, such as antimicrobial, anti-inflammatory, antioxidant, or prebiotic compounds, into fibers or coatings, enabling them to exert therapeutic or protective effects upon contact with the skin (Gulati et al., 2022; Ghosh et al., 2025a) These materials are increasingly being used in dermatology, personal care, medical applications, and everyday wear, where fabrics act as a secondary skin interface. Their functionalities have been especially valuable in preventing infections, modulating inflammatory responses, and improving skin healing (Reta et al., 2024).

Recent years have witnessed an expansion in the use of sustainable, biocompatible materials in bioactive textiles. Natural agents such as chitosan, honey, aloe vera, neem, turmeric, essential oils, and plant extracts have demonstrated broad-spectrum antimicrobial activity against skin pathogens like *Staphylococcus aureus*, *Escherichia coli*, *Pseudomonas aeruginosa*, *Cutibacterium* acnes, and *Candida albicans*, all of which are commonly implicated in skin infections (Szadkowski et al., 2024; Oliveira et al., 2025). These materials have shown great promise in mitigating infection risks while preserving or even enhancing beneficial microbial populations, thereby fostering a healthy skin microbiome.

However, despite their benefits, the precise effects of antimicrobial textiles on the skin microbiome, especially on beneficial species like *Staphylococcus epidermidis*, remain underexplored. This commensal bacterium plays a vital role in skin homeostasis, immune modulation, and pathogen defense. Understanding how bioactive textiles affect both harmful and beneficial skin microbes is crucial for ensuring their safe and targeted application.

To facilitate effective evaluation of these materials, a tiered assessment strategy has been proposed, moving from basic *in vitro* models to more complex, biologically relevant skin models. The one-dimensional (1D) evaluation model begins with classical microbiological assays to evaluate the effects of such materials in skin pathogenic microorganisms, while the final tier includes three-dimensional (3D) advanced skin models that mimic the stratified epidermis/dermis and key microbiota members, enabling a more accurate prediction of *in vivo* behavior (Galvan et al., 2024).

This structured pipeline supports the development of microbiome-compatible textiles and facilitates translation into real-world applications. It enables researchers to fine-tune antimicrobial performance while preserving skin commensals and minimizing inflammation. Moreover, the integration of 3D microbiome-inclusive models ensures more robust safety and efficacy data prior to clinical or commercial deployment.

In summary, bioactive textiles represent a convergence between materials science, dermatology, microbiology, and sustainability, offering innovative solutions that extend from infection control to therapeutic skin care. Their applications range from advanced skin wounds to everyday garments, addressing both health and environmental concerns. Moving forward, the adoption of interdisciplinary approaches, particularly the transition from 1D to 3D evaluation models will be critical to ensure the development of next-generation textiles that are not only effective, safe, environmentally responsible but also skin microbiome-friendly.

## 2 Skin microbiota

**Figure 1:**
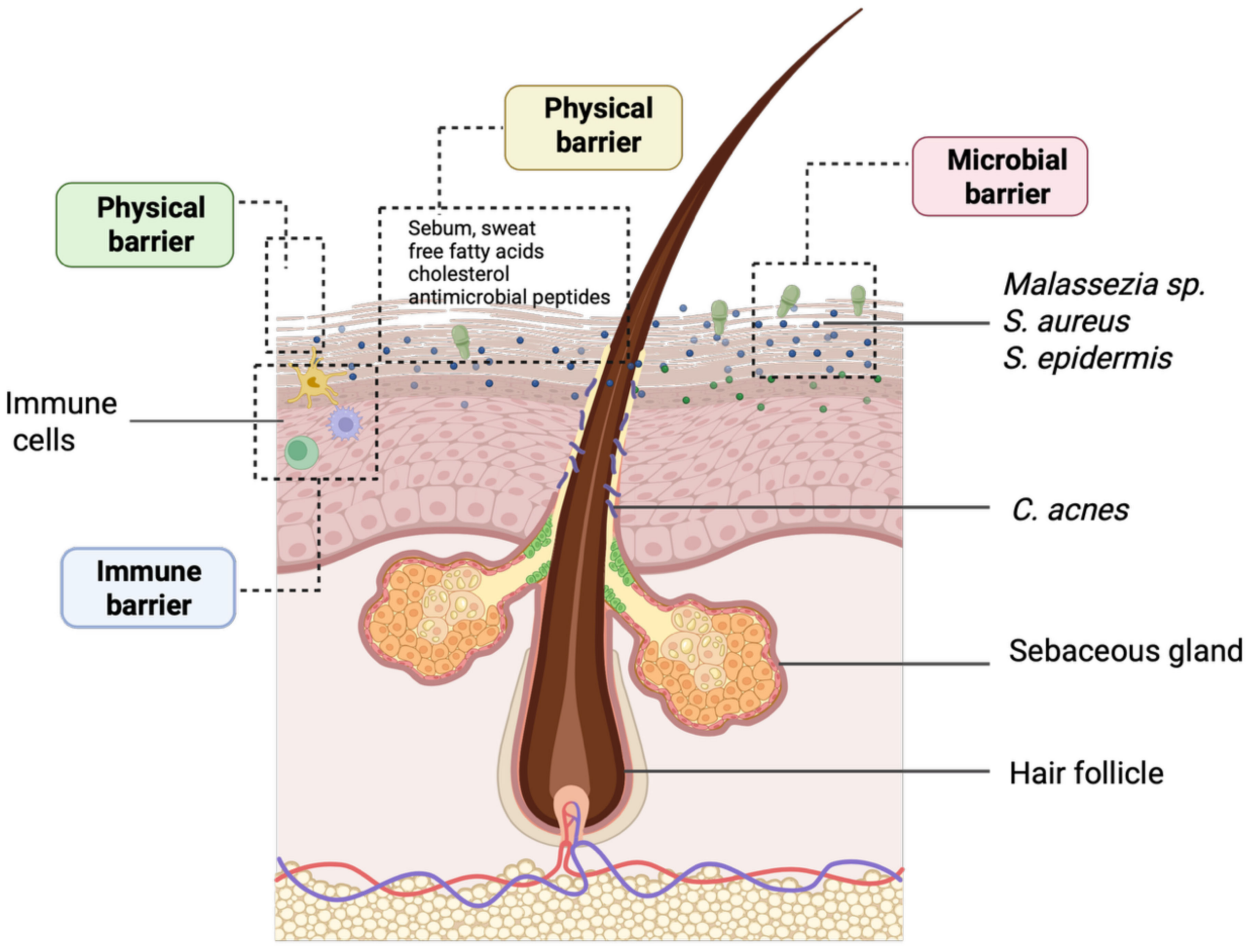
Representative overview of the epidermal barriers and the localization of the most important skin microbiota members. The epidermal barrier protects against pathogens, chemicals, and UV radiation while preventing excessive trans-epidermal water loss. This defense relies on physical, chemical microbiological, and immunological components, largely regulated by a complex lipid network. The image was created in https://BioRender.com.

The human skin functions as a multilayered defense system, comprising four interconnected barriers: physical, chemical, immune, and microbial (Nicolaou and Kendall, 2024) (**Figure 1**). The components operate synergistically to maintain skin homeostasis and prevent the onset of infections, inflammation, and even skin cancers. However, disruptions in any of these systems can compromise the integrity of the others, resulting in skin disease states (Byrd et al., 2018).

The chemical barrier, maintained by its natural acidity and lipid composition, plays a critical role in regulating moisture retention and suppressing microbial overgrowth. Key lipid components, such as ceramides, free fatty acids, and cholesterol, produced by keratinocytes within the stratum corneum, contribute to elasticity, hydration, and defense against pathogen entry (Nicolaou and Kendall, 2024). Simultaneously, keratinocytes establish tight junctions and secrete cytokines and antimicrobial peptides, reinforcing the skin as both a physical and immunological barrier (Zhang et al., 2022). Beyond these structures, the immune defense layer comprises resident innate immune cells, including Langerhans cells and dermal dendritic cells, as well as adaptive immune cells that are recruited in response to stress or infection. Together, these elements coordinate antigen recognition, immune activation, and the development of immunological memory (Coates et al., 2018).

Next-generation sequencing has revealed that the skin microbiome is predominantly composed of Gram-positive bacteria, particularly *Cutibacterium* and *Staphylococcus*, with distribution varying by site (Prajapati et al., 2025) (**Figure 1**). Sebaceous areas typically harbor *Cutibacterium acnes*, whereas moist regions, such as the axillae and elbows, favor *Staphylococcus* and *Corynebacterium* species. Among commensals, *Staphylococcus epidermidis* plays a key protective role by producing antimicrobial peptides, competing with pathogens like *Staphylococcus aureus*, and reinforcing barrier integrity through tight junction protein induction (Koberle et al., 2022).

The fungal community, largely represented by *Malassezia* species, contributes to defense by secreting indoles and other metabolites that inhibit pathogenic fungi (Koberle et al., 2022).

Disruption of the skin microbial ecosystem, through hygiene practices, environmental stressors, or immune dysregulation, can shift commensals toward opportunistic behavior. For example, *Cutibacterium acnes* contributes to acne not by mere overgrowth but through alterations in strain composition (Koberle et al., 2022). Likewise, increased *Staphylococcus aureus* colonization is a hallmark of AD while dysregulated *Malassezia* populations are linked to seborrheic dermatitis (Kreouzi et al., 2025).

This intricate relationship between microbiota and skin barrier function underscores the potential of bioactive textiles that selectively suppress pathogens while preserving beneficial microbes as non-invasive strategies to restore microbial homeostasis.

## 3 Bioactive textiles: a short overview

Bioactive textiles represent a new frontier in material science, distinguished by the incorporation of biologically active components designed to provide therapeutic or protective effects beyond conventional textile properties. These textiles are engineered by incorporating active substances, such as antimicrobial agents, antioxidants, or healing compounds, into the fiber matrix or surface to interact with the user or environment in a purposeful way (Gulati et al., 2022; Szadkowski et al., 2024; Ghosh et al., 2025a). This class of functional fabrics has attracted increasing attention due to its potential in healthcare, personal care, sportswear, and sustainable fashion sectors (Korica et al., 2022; Ghosh et al., 2025b).

Among the most researched bioactivities, the antimicrobial functionality is the most challenging one. This is particularly critical in medical and hygiene contexts, where bioactive textiles serve to prevent infections, reduce pathogen transmission, or manage wound healing.

### 3.1 Fabrication approaches and antimicrobial activity of bioactive textiles

Bioactive textiles are engineered by incorporating functional agents into fabric substrates to endow them with antimicrobial, antioxidant, anti-inflammatory, or other biologically relevant properties. These materials are particularly important in healthcare, personal care, and hygiene applications, where textile surfaces often come in direct contact with human skin and are prone to microbial colonization.

The development of bioactive textiles involves several fabrication and functionalization methods. The most common strategy is finishing, where bioactive compounds are applied onto the fabric surface using techniques such as pad-dry-cure, exhaustion, or spray coating. In this regard, a wide range of antimicrobial agents, both synthetic and natural, have been integrated into textile materials, including triclosan, metals and their salts, quaternary ammonium compounds (QACs), organosilicons, chitosan, essential oils, and plant extracts (Stan et al., 2019; Roman et al., 2020; Hedayati et al., 2021; Julaeha et al., 2021; Mehravani et al., 2021; Garcia et al., 2022; Gulati et al., 2022; Szadkowski et al., 2023). Among these, Ag- and Zn-based salts have shown broad-spectrum antimicrobial activity against diverse microorganisms, such as Gram-positive and Gram-negative bacteria, fungi, viruses, yeasts, and algae (Gopal et al., 2021; Hedayati et al., 2021; Garcia et al., 2022). QACs have also demonstrated notable antimicrobial efficacy, although their activity spectrum is generally narrower than that of metal-based agents (Jennings et al., 2015; Jiao et al., 2017). Recently, plant-derived bioactives (e.g., extracts of neem, aloe vera, eucalyptus, clove, and turmeric) have gained attention for their sustainability and safety profiles. These agents are incorporated into textiles via eco-friendly methods like solvent-free dyeing or embedding during finishing stages (Reta et al., 2024; Oliveira et al., 2025).

Another innovative approach involves immobilization of antimicrobial agents via covalent bonding, which enhances wash durability and minimizes environmental leaching. For instance, chloroxylenol has been covalently bonded to cotton via bifunctional reactive finishes, enabling robust antimicrobial activity even after 20 washing cycles (Reta et al., 2024). Nanoencapsulation is another key method, where many bioactive compounds, (e.g., metals, dyes, essential oils, plant extracts, chitosan) are encapsulated into nanoparticles (NPs) (Roman et al., 2020; Vakayil et al., 2021; Sanchez and Fiscal Ladino, 2023). These are then deposited onto textile fibers to provide sustained release, enhanced fabric bonding, and improved biocompatibility (Costa et al., 2022).

The antimicrobial activity of bioactive textiles is influenced by several parameters, including the adhesion, survival, and proliferation of pathogens on the textile surface, the type and concentration of antimicrobial agents, and the method of their incorporation into the fabric structure. These factors significantly impact both the immediate antimicrobial efficacy and the long-term durability of the textile’s bioactivity (Sanders et al., 2021; Gulati et al., 2022).

Surface characteristics, such as wettability, porosity, and pore size, play a crucial role in bacterial adhesion. For instance, hydrophilic textile surfaces with larger pore volumes promote higher bacterial adherence compared to superhydrophobic surfaces with reduced pore sizes (Hemmatian et al., 2021). Similarly, anti-adhesive coatings, such as poly(l-lysine)-g-poly(ethylene glycol), have demonstrated an over 80% reduction in bacterial attachment on polyester substrates, maintaining their functionality even after 20 wash cycles (Schmidt-Emrich et al., 2016).

Equally important is the method of antimicrobial agent integration, such as physical adsorption, covalent bonding, or NPs embedding, each affecting release profile, durability, and biocompatibility. For example, textiles functionalized with enzymatically bonded nanoAg within dynamic polysulfide networks exhibited >99.99% antibacterial activity, along with excellent wash durability and biocompatibility (Wu et al., 2025).

### 3.2 Mode of action on skin microbiota

Bioactive textiles interact with skin microbiota primarily through their embedded biologically active compounds, which influence the microbial composition and activity on the skin. These materials can both support beneficial microorganisms and suppress pathogenic species, depending on their functional design and the active agents they release (Suellen Ferro de Oliveira and Kekhasharu Tavaria, 2023).

**Figure 2:**
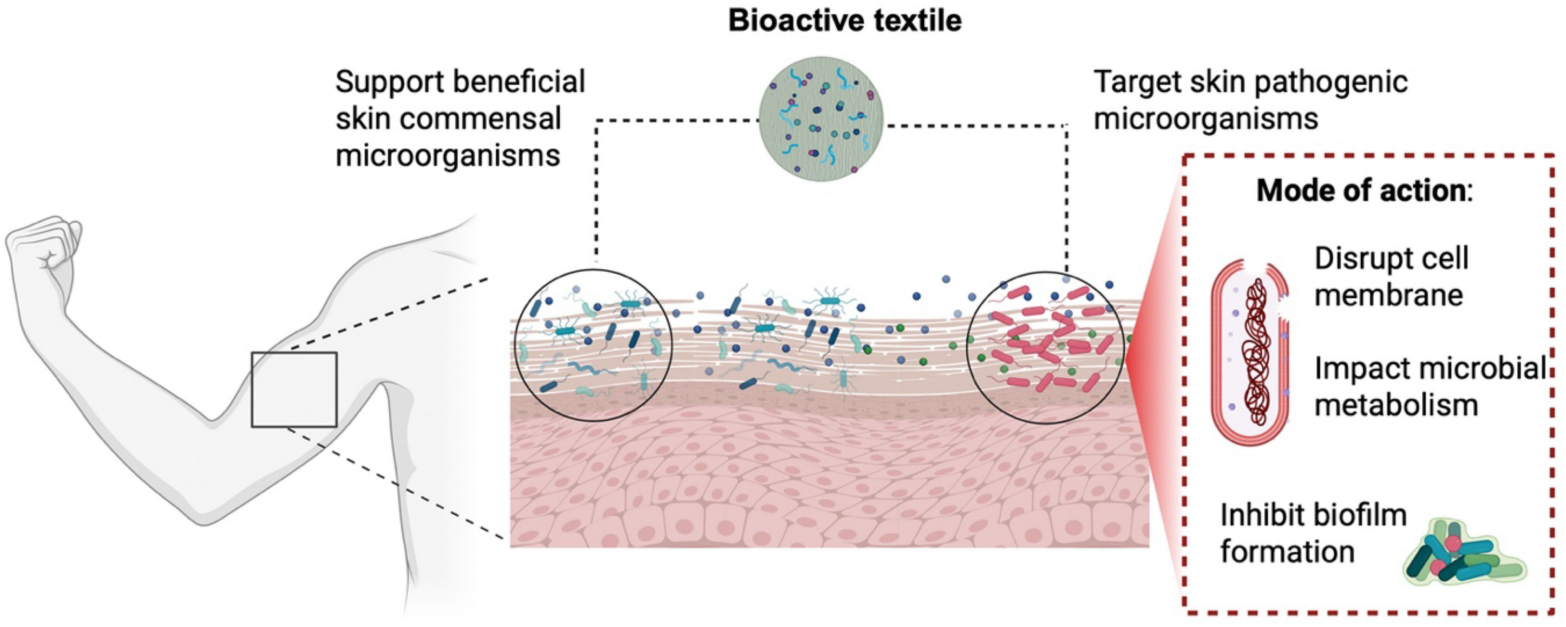
Impact of bioactive textiles with antimicrobial activity in commensal and pathogenic members of the human skin. The image was created in https://BioRender.com.

Antimicrobial action is typically achieved through the integration of metal NPs (e.g., Ag, ZnO), chitosan, plant-based compounds, or biosurfactants. These agents act by disrupting microbial membranes, interfering with metabolism, or inhibiting biofilm formation (Ghosh et al., 2025b) (**Figure 2**). Natural antimicrobials, such as essential oils or polyphenols, are increasingly favored due to their biodegradability and lower toxicity compared to synthetic alternatives (Ghosh et al., 2025b).

In parallel, sustainability concerns are driving innovation toward eco-friendly and circular materials. Researchers have demonstrated the successful use of agro-industrial waste (e.g., grape pomace, peanut skins, pomegranate peels) as sources of natural dyes and bioactives for textiles, enabling the valorization of biowaste while imparting antimicrobial and antioxidant effects (Lopes et al., 2025).

Moreover, therapeutic textiles with antimicrobial activity have shown promise in managing skin dysbiosis by integrating antimicrobial and antioxidant functions to reduce inflammation and support a healthy microbiome (de Oliveira and Tavaria, 2024). However, standardized models for microbiome safety assessment, along with comprehensive clinical studies, are still necessary to explore and validate the long-term effects of these materials.

In this line, a growing area of concern and innovation is the interaction between bioactive textiles and the skin microbiome. While many antimicrobial fabrics are effective against pathogens, they may also harm beneficial microbes such as *Staphylococcus epidermidis*, which play a vital role in skin homeostasis and immune regulation. This has led to increasing emphasis on developing microbiome-safe textiles that preserve or even promote commensal microbial populations while selectively targeting pathogens (Suellen Ferro de Oliveira and Kekhasharu Tavaria, 2023).

Prebiotic-infused textiles represent another approach, where materials contain substances that selectively support beneficial microbes. For example, short-chain fructo-oligosaccharides (scFOS) applied to skin models or incorporated into biomaterials have been shown to promote *Staphylococcus epidermidis* while inhibiting *Cutibacterium acnes* and *Staphylococcus aureus (* Le Bourgot et al., 2022 *)*. This strategy aligns with efforts to design microbiome-friendly products that enhance skin barrier function while minimizing inflammation and opportunistic infections.

Multifunctionality is also a central theme in the field. Emerging bioactive textiles are not limited to one function but often combine antimicrobial, antioxidant, anti-inflammatory, and moisture-regulating properties. For instance, cellulose-based wound dressings enriched with ascorbic acid or other antioxidants have demonstrated not only strong biocompatibility but also enhanced healing capabilities, highlighting the therapeutic potential of such textiles (Firmanda et al., 2024).

## 4 Evaluation strategies for antimicrobial bioactive textiles: a 1D conceptual framework

To facilitate the systematic development and assessment of antimicrobial bioactive textiles, a 1D evaluation model has been extensively used. In this context, a 1D model represents a linear, stepwise conceptual framework in which textile materials are assessed progressively through increasingly complex and biologically relevant assays. This classic 1D pipeline begins with basic antimicrobial screening and advances through mechanistic characterization, functional validation, and ultimately biological relevance.

The first stage of testing the antimicrobial activity of bioactive textiles on the 1D model involves traditional *in vitro* antimicrobial screening, using standardized microbiological assays designed to evaluate their direct effects on clinically relevant pathogens (Hedayati et al., 2021; Garcia et al., 2022; Mirza et al., 2023; Liu et al., 2024). These include agar diffusion (to assess inhibition zones), colony-forming unit (CFU) counting, and broth dilution techniques (Ivankovic et al., 2022). These methodologies provide both qualitative and quantitative insights, serving as a crucial step in screening the antimicrobial performance of such materials. These assays evaluate microbial growth inhibition under conditions where the microorganism is in direct contact with the textile material, thus enabling a comprehensive evaluation of the material’s inhibitory potential against common pathogens such as *Staphylococcus aureus*, *Escherichia coli*, *Klebsiella pneumoniae*, and *Candida albicans (* dos Santos et al., 2021 *)*.

Following initial screening, materials demonstrating antimicrobial activity undergo advanced mechanistic assays to further elucidate antimicrobial mechanisms. These include live/dead fluorescence microscopy to distinguish viable from non-viable cells, metabolic activity assays (e.g., 3-(4,5-dimethylthiazol-2-yl)-2,5-diphenyl tetrazolium bromid - MTT, resazurin reduction), adenosine triphosphate (ATP) quantification to assess microbial viability, and quantitative real-time PCR (qPCR) to quantify microbial DNA load. These approaches are particularly useful for evaluating the impact of bioactive textiles on biofilm formation and persistence, addressing a critical challenge in chronic skin infections and antimicrobial resistance (AMR) (dos Santos et al., 2021; Oliveira et al., 2025).

In the third stage of the pipeline, functional performance is evaluated beyond planktonic microbial inhibition. Biofilm formation assays assess whether the textile can inhibit microbial adhesion and colonization (dos Santos et al., 2021). Antioxidant properties are measured using 2,2-diphenyl-1-picrylhydrazyl (DPPH) and 2,2’-azino-bis(3-ethylbenzothiazoline-6-sulfonic acid) (ABTS) radical scavenging assays to determine the textile’s ability to neutralize reactive oxygen species (ROS) (Oliveira et al., 2025), which are involved in inflammation and delayed healing. Such multifunctionality enhances the applicability of antimicrobial textiles in clinical and daily-use settings.

However, the traditional antimicrobial evaluation typically target pathogens implicated in skin wound infections and hospital cross-infections, namely Gram-positive bacteria (e.g., *Staphylococcus aureus*, *Staphylococcus pyogenes*), Gram-negative bacteria (e.g., *Escherichia coli*, *Pseudomonas aeruginosa*, *Klebsiella pneumoniae*), and opportunistic fungi such as *Candida albicans* (Smith et al., 2020; dos Santos et al., 2021; Garcia et al., 2022; Vojnits et al., 2024). Given the increasing concern over AMR, recent studies also incorporate multi-drug resistant (MDR) strains, such as methicillin-resistant *Staphylococcus aureus* (MRSA), vancomycin-resistant *Enterococcus* (VRE), and carbapenem-resistant *Pseudomonas aeruginosa*, to assess the efficacy of bioactive textiles in combating persistent infections (Vojnits et al., 2024).

In this sense, many antimicrobial textiles, particularly those impregnated with Ag, copper, ZnO, antibiotics, and anti-inflammatory drugs, have been shown to reduce skin-associated pathogens in conventional *in vitro* antimicrobial assays (Garcia et al., 2022; Markovic et al., 2022; Liu et al., 2024). For example, cotton fabrics coated with silica NPs containing both an anti-inflammatory drug (ibuprofen) and an antibiotic (levofloxacin or norfloxacin) designed for wound healing, exhibited significant antibacterial activity against *Staphylococcus aureus* using standard agar diffusion assay (Liu et al., 2024). Similarly, polyamide fabrics incorporating zinc oxide NPs exhibited significant antibacterial effects against *Staphylococcus aureus* and *Escherichia coli*, supporting their potential use in medical and hygiene applications (Garcia et al., 2022).

In another example, antibacterial nanocomposite textiles were developed by coating cotton/peat blend fabrics with chitosan and Cu_2_O/CuO nanostructures with gallic acid serving as a reducing agent (Markovic et al., 2022). The treated fabrics showed enhanced antibacterial activity against *Staphylococcus aureus* and *Escherichia coli*. Likewise, cotton fabrics using bioactive formulations combining chitosan and thyme essential oil, along with mineral fillers like silica, ZnO, and TiO_2_, presented strong antimicrobial activity against various microorganisms, including *Staphylococcus aureus*, *Escherichia coli*, and *B. subtilis (* Szadkowski et al., 2023 *)*. In addition to their antimicrobial properties, these bioactive fabrics also demonstrated flame-retardant activity and biodegradability. According to the authors, such multifunctional bioactive textiles hold promising for advanced applications in military and medical context.

Although synthetic antimicrobial agents are known for their potent and often broad-spectrum activity, their incorporation into textile materials raises significant environmental and health concerns. Many of these compounds are linked to potential human toxicity and ecotoxicological risks, thereby posing serious sustainability challenges. For example, during laundering, these agents can leach from the textile fibers and enter aquatic ecosystems, where they may disrupt microbial communities and exert toxic effects on aquatic organisms (Bibi et al., 2024). Such unintended environmental release not only negatively impact the ecological balance but also raises concerns about the widespread use of synthetic antimicrobials in textile materials applications.

These limitations highlight the growing need for safer and more sustainable antimicrobial alternatives that are both biodegradable and biocompatible. Natural agents, including biopolymers, plant-derived compounds, and green-synthesized NPs, are emerging as promising solutions, offering antimicrobial properties while minimizing environmental and health-related risks (Costa et al., 2018; Stan et al., 2019; Mouro et al., 2020; El-Tarabily et al., 2021; Soroh et al., 2021; Choi and Park, 2023; Freitas et al., 2024; Pallottelli et al., 2024). Among these, chitosan, honey, and plant-based products, namely aloe vera, essential oils, and various plant extracts, have demonstrated broad-spectrum antimicrobial activity against pathogenic bacteria and yeasts such as *Bacillus subtilis*, *Escherichia coli*, *Pseudomonas aeruginosa*, *Cutibacterium acnes*, *Staphylococcus aureus*, and *Candida albicans* when incorporated into textile materials.

For example, cotton fabrics treated with a biosolution containing propolis and honey, with or without potassium alum as a mordant, exhibited significant antibacterial effects against *Staphylococcus aureus*, *Bacillus subtilis*, *Cutibacterium acnes*, and *Escherichia coli (* Freitas et al., 2024 *)*. In addition to their antimicrobial properties, these textiles showed potent antioxidant activity, achieving over 90% ABTS radical scavenging. These findings emphasize the potential of beehive-derived products, such as propolis and honey, as multifunctional natural agents for daily personal care applications. Their integration into reusable products like face masks not only enhances antimicrobial and antioxidant protection but also aligns with the principles of sustainability and circular product design.

Another example investigated cotton fabrics dyed with *Clostridium obtusa* extract in the presence of various mordants, particularly aluminum and copper (Choi and Park, 2023). These bioactive fabrics demonstrated not only strong antibacterial activity against *Staphylococcus aureus* and *Klebsiella pneumoniae*, two common pathogens frequently isolated from healthcare workers’ uniforms, but also higher antibacterial activity against the MRSA super bacteria. Based on these results, the authors propose the integration of *Clostridium obtusa*-dyed textiles into hospital environments, including patient garments, bed linens, and healthcare uniforms, to reduce cross-infection risks and reinforce infection control strategies.

Despite the promising antimicrobial performance of some natural agent-based bioactive textiles, a critical limitation remains: their reduced durability and diminished effectiveness after repeated laundering (Babu and Ravindra, 2015). Therefore, there is a need for further research focused on enhancing wash fastness and long-term functional stability to enable broader, real-world adoption in clinical and personal care contexts.

Bioactive textiles with anti-biofilm properties represent another growing area of interest, particularly in medical and hygiene applications. These materials are typically functionalized with antimicrobial agents capable of inhibiting the adhesion, colonization, and proliferation of biofilm-forming microorganisms, namely *Staphylococcus aureus*, *Escherichia coli*, *Klebsiella pneumoniae*, and *Candida albicans (* dos Santos et al., 2021; Mirza et al., 2023 *)*. This is especially critical in healthcare settings, where biofilms can contribute to persistent skin infections and reduce the effectiveness of conventional treatments. Among the various antimicrobial functions explored in bioactive textiles, antibiofilm activity is particularly important for skin wound applications, where preventing microbial biofilm formation is essential to promote healing and reduce the risk of chronic infection.

**Figure 3:**
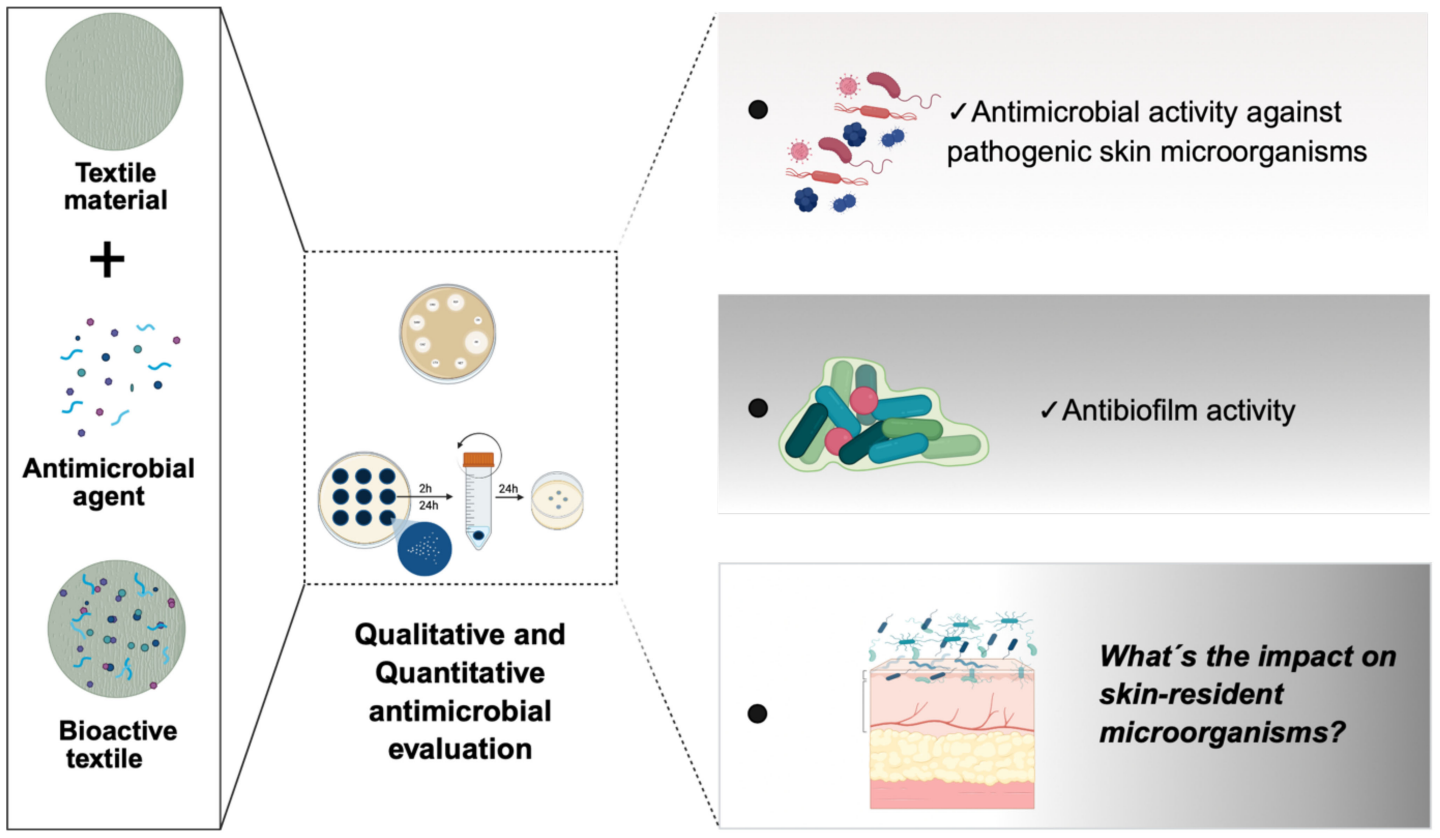
From design to microbiome impact - Evaluating bioactive textiles for skin applications. Left panel (design of bioactive textiles): Textile materials are functionalized with antimicrobial agents to develop bioactive textiles aimed at targeting pathogenic skin microorganisms. Center panel (assessment of antimicrobial activity): Standard antimicrobial assays are conducted to evaluate the antimicrobial properties of the bioactive textiles. These include disc diffusion, time-kill kinetics, and microdilution assays, performed under various conditions and different time points to assess their performance against microbial growth. Right panel (evaluation of biological impact): Key outcomes assessed include: i) Antimicrobial activity against pathogenic skin microorganisms; ii) Antibiofilm activity, reflecting the ability of bioactive textiles to inhibit or disrupt microbial biofilm formation; iii) Impact on skin-resident microorganisms (microbiome safety): a critical underexplored area, focusing on the preservation of beneficial commensal skin microbiota. The image was created in https://BioRender.com.

While existing studies have provided valuable insights into the ability of bioactive textiles to inhibit pathogenic microorganisms, as well as antibiofilm activity, their impact on the skin’s commensal microbiota remains largely unexplored (**Figure 3**). Limited information is available regarding the effects of such materials on *Staphylococcus epidermidis*, a dominant member of the human skin microbiome that plays a critical role in maintaining skin homeostasis. This knowledge gap raises concerns about the potential unintended consequences of antimicrobial textiles on *Staphylococcus epidermidis* and other beneficial skin microbes. Addressing this issue is essential for the development of microbiome-friendly textiles that combine effective antimicrobial performance with the preservation of skin microbial balance.

## 5 Transitioning bioactive textiles to 2D skin models

**Figure 4:**
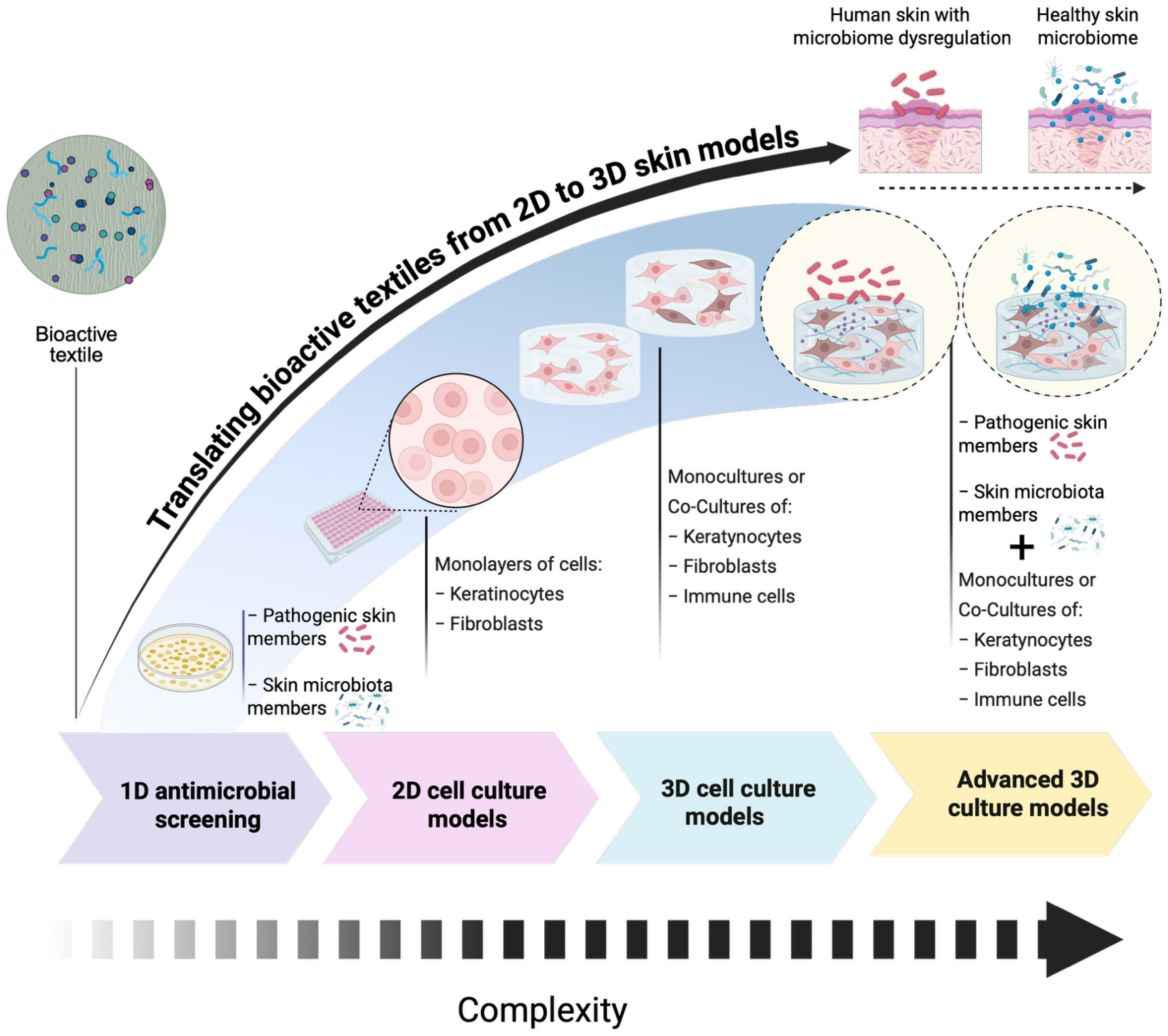
Schematic representation of the progression of *in vitro* models used to evaluate antimicrobial strategies of bioactive textile materials. The models evolve in complexity from left to right using 1D antimicrobial screening (simplified systems with pathogens in indirect or direct contact with bioactive textiles). Main limitation: These models do not include host cells, providing limited insight into host–pathogen interactions. 2D cell culture: Involves monolayers of keratinocytes and/or fibroblasts grown on flat surfaces. These models enable initial screening of host responses but lack the structural and cellular complexity of skin. 3D cell culture: Incorporates 3D organization of cells, such as fibroblasts and keratinocytes, in monocultures or co-cultures. These models better mimic the skin’s architecture and can include immune cells for enhanced relevance. Advanced 3D culture: Represents the most physiologically relevant *in vitro* skin models. These systems support the co-culture of keratinocytes, fibroblasts, and immune cells in a 3D matrix and can be challenged with skin-relevant pathogens (e.g., bacteria or fungi). They allow detailed study of host–microbe interactions and antimicrobial efficacy under near-physiological conditions. The image was created in https://BioRender.com.

Following initial antimicrobial screening using 1D microbiological assays, some bioactive textiles are typically assessed using 2D cell culture models (**Figure 4**). 2D models have been useful to evaluate the biocompatibility of bioactive textile materials, particularly when intended for direct contact with human skin. These 2D models typically consist of monolayers of human skin keratinocytes or human dermal fibroblasts, which provide fundamental insights into cell viability, proliferation, metabolic activity, ROS, and early-stage biocompatibility (Hassan et al., 2024; Pallottelli et al., 2024; Oliveira et al., 2025).

At this stage, some studies were dedicated to the evaluation of bioactive textiles antimicrobial activity with preliminary cytotoxicity assessments (Hassan et al., 2024; Pallottelli et al., 2024). In terms of biocompatibility, most antimicrobial textiles have shown no cytotoxic or moderate cytotoxic effects when tested using either indirect or direct contact with 2D human skin cell models. For example, cotton fabric loaded with Ag NPs, combined with ketoconazole and β-cyclodextrin, exhibited potent antifungal and antibacterial activity against key skin pathogens, including *Candida albicans*, *Aspergillus niger*, *Staphylococcus aureus* and *Escherichia coli* (Hedayati et al., 2021). The cytotoxicity was evaluated via an indirect method using a monolayer of human skin fibroblasts, revealing no cytotoxic effects after 24h of incubation. According to the authors, these findings support the safety of the bioactive textile materials for skin contact and highlight its potential for dermatological and personal care applications. Likewise, cotton gauze coated with a dual layer system of Poly (vinyl alcohol) and chitosan containing extracts of *Agrimonia eupatoria L*. demonstrated effective inhibition of *Staphylococcus aureus* and *Pseudomonas aeruginosa* growth (Mouro et al., 2020). Cytotoxicity was assessed using a direct contact method with a human dermal fibroblast monolayer, which showed no cytotoxic effects after 1, 3, and 7 days of incubation, indicating sustained biocompatibility over time.

Although these studies demonstrate the feasibility of developing bioactive textiles with effective antimicrobial properties against well-known skin pathogens and acceptable biocompatibility, they often fail to assess the broader impact of such materials on the skin’s resident microbiota members. Specifically, there is a lack of investigation into whether these materials selectively inhibit pathogenic microorganisms or unintentionally disrupt commensal microbial populations that are essential for skin health. Furthermore, their potential to modulate the balance between pathogenic and beneficial microbes, namely in the context of skin conditions pathological conditions correlated with microbiome dysregulation remains largely unexplored.

Despite these gaps, bioactive textiles targeting the skin microbiome dysregulations, namely AD, are frequently advanced to clinical trials after only minimal cytotoxicity testing, typically limited to short-term assays on 2D cell cultures (de Oliveira and Tavaria, 2024; Hassan et al., 2024). This practice highlights a critical shortfall in preclinical validation and highlights the need for more comprehensive evaluation of their impact on the human skin microbiome, particularly through the extensive use of advanced *in vitro* models such as 3D reconstructed human skin, prior to clinical translation.

## 6 3D skin models: essential tools for evaluating the impact of bioactive textiles on the skin microbiome

Despite their widespread use, conventional 2D culture systems are unable to fully capture and recapitulate the structural and physiological complexity of human skin. This limitation hinders the comprehensive evaluation of key parameters failing to predict critical functional outcomes, including tissue penetration, barrier integrity, cell-cell, cell-microbe, cell-material interaction, microbiome impact and modulation, immune responses, wound healing capacity, and tissue regeneration. Therefore, 2D tissue models are considered unreliable pre-clinical testing tools. Indeed, to ensure the safe and effective clinical translation of bioactive materials, it is essential to first accurately replicate the native skin microenvironment, particularly its cellular composition, structural organization, and dynamic biological interactions. In this context, 3D human skin models have emerged as physiologically relevant platforms that more accurately mimic key properties of the human skin, including its architecture and functionality (Poumay and Coquette, 2007; Flaten et al., 2015; Niehues et al., 2018; Villata et al., 2024). Moreover, the bottom-up approach underpinning the design of such multi-component 3D *in vitro* models makes them suitable candidates for the investigation of pathological conditions and for testing the safety and efficacy of innovative biomaterials and therapies. For instance, the combination of healthy skin models with cells from the immune systems, such as pro-inflammatory macrophages, could become a reliable pathological platform for wound healing-related studies being able to mimic the prolonged inflammation and oxidative stress proper of chronic skin wounds. On the other hand, the contamination of such 3D models with specific bacterial strains in their planktonic or multi-species form leads to the development of pathological platforms suitable for the investigation of the efficacy of innovative antimicrobial therapies.

**Figure 5:**
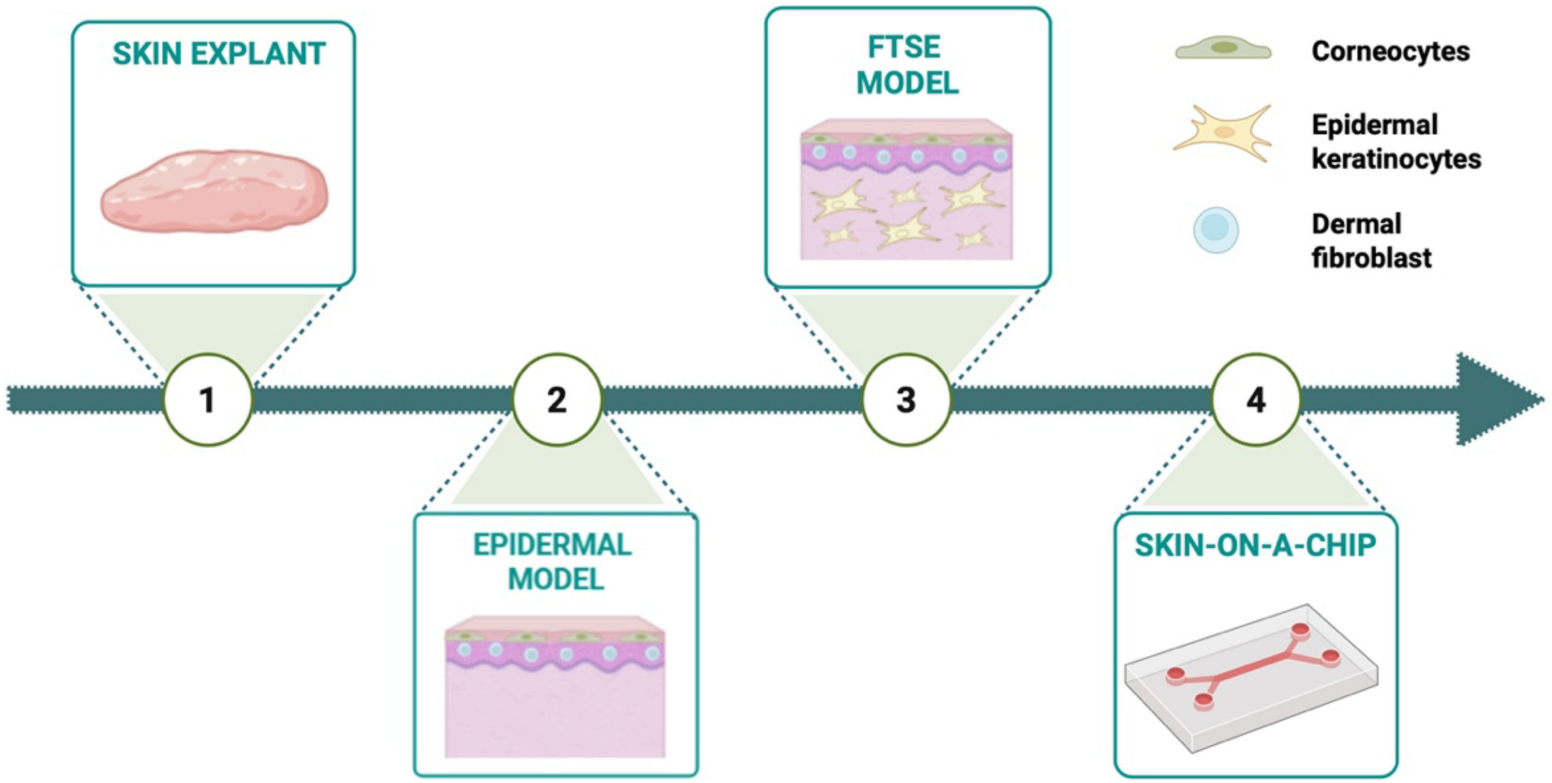
Evolution of 3D *in vitro* skin model from skin explants to skin-on-a-chip systems. The Figure was created in Biorender.

**Figure 5** schematically represents the evolution of 3D *in vitro* skin models from the easiest skin explant models to the advanced bioengineered skin-on-a-chip platforms. Moreover, particular attention will be focused on models specifically designed for wound healing- and microbiome-related applications.

### 6.1 Skin explants

The skin is the largest organ of the human body, playing fundamental roles such as serving as a protective physical barrier, controlling thermoregulation, providing sensation, maintaining homeostasis, and supporting immunity. It is composed of three layers: epidermis, dermis, and subcutis, and contains different cell phenotypes (Maggiotto et al., 2025). To study its complex structure and interaction with external agents, skin explants, i.e., native tissue portion from a donor, are considered the most realistic 3D skin models.

Most commonly, skin explants come from plastic surgery or cadavers, and in both cases, proper ethical approval is needed for their use in research (Abd et al., 2016). Generally, skin explants are derived from abdominal, breast, or back skin because they are the most abundant donor sites. Two types of skin explants can be distinguished: full-thickness and partial-thickness skin explants. The former contains both epidermal and dermal layers, and it is characterized by all skin cell types, guaranteeing an accurate representation of their physiological and functional interconnections. On the other hand, it is affected by some limitations such as loss of the vascular and nervous systems. Although these disadvantages exist, full-thickness skin explants are considered physiologically relevant models for studying the mechanisms contributing to skin disease (Mathes et al., 2014). Differently, the partial-thickness skin explant is composed of the full epidermidis and a portion of the underlying dermis. However, this model captures only part of the complexity of human skin, as endothelial cells, melanocytes, and Langerhans cells are gradually lost under culture conditions over time.

Irrespective of the type of skin explants, they are considered promising tools to evaluate the efficacy and skin absorption of personal care products, as well as their effects on skin infections, metabolism, immune responses, melanogenesis, and irritation. The relevance of these models is further highlighted by several studies that have employed skin explants in various experimental contexts, as outlined below.

For instance, Eberlin et al. used the skin explant to evaluate various skin disorders (i.e., photoaging, skin barrier disorders, AD), and permeation of substances through the skin using immunofluorescence, immunoassay, and omics platforms to study the interaction between cutaneous tissue and environmental factors (Eberlin et al., 2020). Another example employed an *ex vivo* wound model based on full-thickness human skin to evaluate the polyvinylpyrrolidone foils and nanofiber mats as a ciprofloxacin delivery system for treating infected wounds (Rancan et al., 2019). More recently, a 3D ovine skin explant model was exploited to investigate anaerobic infection by *Dichelobacter nodosus* (Maboni et al., 2017). The model preserved tissue viability for up to 28 h, showed bacterial invasion of the epidermis, and triggered an inflammatory response, demonstrating its potential for studying anaerobic skin infections.

Despite skin explants being closer to the *in vivo* environment, they are characterized by high inter-donor variability, availability, and biological limitations. Indeed, the cutaneous absorption response is strongly influenced by the anatomical site of collection, due to stratum corneum thickness, hydration, and lipid composition (Abd et al., 2016).

### 6.2 Epidermal models

The human epidermis is a self-repairing barrier that separates our internal body from the external environment. Its main role is to protect the body against dehydration, nutrient loss, and physical, chemical, and biological hazards. Concerning cell phenotypes, keratinocytes are the main cells involved in the production and maintenance of the epidermal barrier. During the progressive maturation, keratinocytes change their morphology from a cuboidal shape located in the basal layer to a squamous morphology in the cornified layer. Other morphological changes include the development of a prickle-cell shape in the spinous layer and the keratohyalin granules, within the granular layer beneath the cornified barrier. Other cell types present in the epidermis are Merkel cells and Langerhans cells.

Understanding the cell population and the differentiation program of epidermal keratinocytes toward skin barrier formation, as well as the impact of external agents and drugs on the skin, requires the development of reliable epidermal models (Poumay and Coquette, 2007). However, such models partially reproduce the skin being composed of only the epidermal layer and not considering the dermal layer and immune cells. Commercially, several epidermal models are available on the market such as EpiSkin^®^, SkinEthic^®^, EpiDerm^®^ which are useful to study skin irradiation, corrosion, penetration, UV damage, bacterial adhesion, and epidermal permeability (Flaten et al., 2015; Pupovac et al., 2018). Poumay et al. developed a fully differentiated cultured epidermis anchored on a polycarbonate filter (Poumay et al., 2004). The obtained model successfully revealed the characteristic morphology with basal, spinous, granular, and cornified epidermal layers, as confirmed by histological analysis, immunofluorescent staining and electron microscopy. Therefore, the proposed model showed promises for the investigation of cell biology, toxicological tests, and drug skin absorption mechanisms.

In fact, epidermal models represent a significant *in vitro* tool for understanding skin physiology, irritation, and permeability. They represent a simplified approximation of human skin structure due to the absence of the dermal layer, which limits their physiological relevance. A more realistic representation of human skin is achieved through more complex models, as described in the following sections.

### 6.3 Full-thickness skin equivalents

Full-thickness skin equivalents (FTSEs) are 3D *in vitro* models that more realistically reproduce both epidermis and dermis layers, obtained by the maturation of keratinocytes and fibroblasts, respectively. Specifically, the engineering of FTSEs encompasses (i) fibroblasts seeding followed by maturation of the dermal layer; (ii) keratinocytes seeding followed by maturation of the epidermal layer and, (iii) establishment of the air-liquid interface (ALI) to allow epidermal layer stratification and keratinization (Moon et al., 2021; Villata et al., 2024).

FTSEs can be classified in scaffold-free and scaffold-based 3D models: in the first case, skin cells are layer-by-layer seeded on a support without the use of any additional materials and allowed to deposit their own extracellular matrix (ECM) (Michel et al., 1999; Liu et al., 2013); in the latter case, cells are embedded in hydrogel-based formulations which function as an hydrated environment resembling the native ECM (Reuter et al., 2017; Casale et al., 2018). For instance, to engineer the dermis, fibroblasts are often seeded in hydrogels prepared from polymers of natural origin, such as collagen/fibrin hydrogel (Bauer et al., 2022). Concerning the epidermis, the development is more complicated due to its several distinct layers.

Based on the reconstruction method of the dermal skin layer, FTSE models can be also classified into three categories: collagen-based, de-epidermized dermis, and self-assembled skin substitutes (Moon et al., 2021). The first one involves seeding epidermal keratinocytes on dermal matrix composed of collagen and dermal fibroblasts. Collagen gel not only serves as support but also supplies nutrients and guarantees cell-to-cell and cell-to-matrix interactions. This model ensures excellent biocompatibility and cell adhesion. However, it shows limited mechanical strength and a short service life. The second model consists of decellularized human skin, used as a dermal substitute that supports keratinocyte attachment and proliferation. The last model consists of keratinocytes and fibroblasts without the addition of exogenous extracellular matrix materials. Dermal fibroblasts are cultured until they reach confluence, secreting their extracellular matrix to form a dermal sheet. Keratinocytes are then seeded on top of these stacked dermal sheets, where they undergo differentiation and cornification. Through the addition of different cell types inside FTSEs, it is possible to investigate the role of these cells in skin health and skin disease (Pupovac et al., 2018).

Mertsching et al. demonstrated the versatility of this model across various applications. Firstly, they investigated the irritating effects of different tested compounds on FTSE (Mertsching et al., 2008). Secondly, they employed the skin equivalent as an *in vitro* tumor model by introducing various tumor cell lines. Additionally, they utilized the model as a disease platform by culturing bacteria on the FTSE to simulate infections, and as a wound model by mechanically creating scratches on the surface.

Therefore, considering their 3D organization and composition, FTSE models are more reliable than the previously described model. However, they also present some disadvantages, such as high costs, limited suitability for long-term culture, and the absence of a vascularization system.

### 6.4 Skin-on-a-chip platforms

A promising alternative approach in the design of advanced 3D skin models is the integration of microfluidic systems. Conventional 3D constructs fail to accurately replicate human skin, not only because they consist of a limited number of cell types, but also due to the lack of a vascularized network (Sun et al., 2023). The absence of blood vessels limits the perfusion of nutrients and drugs, leading to results that do not accurately reflect real physiological conditions. Dynamic culture allows the recreation of a more physiologically controlled environment, defining physical and biochemical parameters as flow, forces, and chemical gradient (Risueno et al., 2021).

In this line, Mohamadi et al. demonstrated that a microfluidic skin system can better replicate a physiological environment by comparing static and dynamic culture conditions in terms of mechanical strength, water adsorption, skin morphology, gene expression, and biopsy longevity (Mohamadali et al., 2023). Regarding the architecture of microfluidic systems, two different strategies have been implemented:

- transferred skin-on-a-chip.
- *in situ* skin-on-a-chip.

In the first approach skin fragments derived either from *ex vivo* human skin biopsies or *in vitro* generated human skin equivalents are introduced in the microfluidic chip systems and perfused by a microfluidic channel below the tissue construct (Risueno et al., 2021). In the second strategy, skin tissue models are directly generated within the chip due upon maturation of seeded cells. In this configuration the microfluidics system not only perfuses the nutrients but also supports the maturation of the tissue. These models equipped with a dynamic perfusion system, better mimic physiological conditions, enabling realistic studies of absorption, diffusion, efficacy, and potential toxicity (Sun et al., 2023).

For this reason, skin-on-a-chip platforms have found relevant applications in the pharmaceutical and cosmetic field, as they enable the evaluation of drugs or products in a controlled microenvironment (Risueno et al., 2021). For instance, Quan et al. developed a reliable tissue model designed for toxicology studies and drug testing in the context of bacteria-infected, damaged skin (Quan et al., 2022). They fabricated an interface-controlled skin-on-chip integrating cutaneous extracellular matrix and cells within a microfluidic system featuring mechanical stimulation. The combination of a perfusion system and an air–liquid interface enabled the development of an *in vitro* skin model with enhanced morphological maturation and improved barrier function. After the maturation of the healthy skin model, bacteria were seeded on the top establishing a pathological model. The authors treated the model with dexamethasone and polyphyllin H, which demonstrated a significant repair effect on the skin barrier.

The integration of the skin model into a microfluidic system allows for a better simulation of real physiological conditions and enables the development of an automated platform capable of simultaneously analyzing multiple samples under different experimental conditions.

Furthermore, one major advantage of this technology lies in the possibility of incorporating biosensors to monitor skin status and drug delivery in real time. In conclusion, skin-on-chip represents a powerful tool for studying the biological mechanisms, both healthy and pathological, that affect this organ.

### 6.5 Case study applications of 3D *in vitro* skin models

#### 6.5.1 Wound healing models

Wounds refer to skin damage characterized by cuts, punctures, burns, or other conditions. They are categorized as either acute or chronic depending on their healing duration and progression (Ahmad, 2022). Engineering acute or chronic wound models represent a promising strategy for studying and understanding the wound healing pathway and preclinically testing innovative biomaterials and therapies.

In this regard, several grafts are commercially available on the market to study the healing of wounds, such as Hyalograft 3D, Apligraf and TissueTech Autograft system. On the other hand, models such as EpiDermFT™, Phenion^®^ FT Model, and StrataTest^®^ are used for 3D wound healing assays (Stamm et al., 2016). To create a wound model, the process begins with a skin model consisting of a dermal layer composed of fibroblasts and an epidermal layer in which keratinocytes are cultured. The epidermal layer is exposed to air to achieve a structure that closely resembles the physiological one. Once a mature skin model is developed, artificial wounds mimicking burn or excisional wounds are performed. Concerning burn wounds, Schneider et al. created a highly standardized *in vitro* 3D epidermal burn model derived from primary epidermal keratinocytes (Schneider et al., 2021). Healthy epidermal models were cultured for 12 days before being burned through a metal rod at room temperature. The wound model was cultured *in vitro* up to 14 days, then treated using drug-based therapies. Specifically, this model was exploited to study the physiological wound healing pathway up to 14 days and to test the safety of pharmacological agents. Although this approach is widely used and efficient, it is associated with high variability in terms of shape and size of resulting wounds. In this regard, Javid et al. proposed an alternative approach, using a 3D printer to create a more reliable tool for producing standardized and reproducible burn wounds characterized by controlled geometry (Tabatabaei and Javid, 2024).

Regarding excisional wounds, lesions are mechanically induced using scalpels and mashers (Stamm et al., 2016). Silva et al., 2025 developed a bioengineered skin wound model by using needles to puncture the skin structure. By modulating needle length, penetration force, and angle, this method allowed precision, and control over the depth, size, and shape of the wound (Salazar Silva et al., 2025). The proposed protocol was controlled, reproducible, standardized, versatile, and scalable for analyzing wound healing cascade and potential treatments. The onset of chronic wounds is often associated with bacterial infections.

Bioengineered wound models are commonly employed for the evaluation of antibacterial agents. In this line, a study investigated new antimicrobial therapeutics and host-pathogen interactions on mechanically wounded human skin models infected with *Staphylococcus aureus* and *Pseudomonas aeruginosa* (Wiegand et al., 2024). This research used the model to investigate the efficacy of silver-containing and polyhexamethylene biguanide-containing wound dressing. Additionally, Andersoon et al. studied the effect of antimicrobial compounds and formulations on *ex vivo* porcin skin model (Andersson et al., 2021). The authors proposed a biologically relevant platform which represented a bridge between the *in vitro* and the *in vivo* model. The model featured a natural ECM and realistic tissue architecture that faithfully replicated the wound bed microenvironment. Infection in the model was induced by introducing two bacterial species commonly involved in wound healing processes. Using scanning electron microscopy (SEM), the authors observed biofilm formation, closely resembling the physiological condition. This model presented itself as a valuable tool for studying the penetration efficacy of topical formulations and materials, including bioactive textiles.

Although 3D wound models represent a valid alternative to *in vivo* testing, they still offer only an approximate representation of physiological reality. Ideal models should accurately replicate the pathological phenotype. It would be beneficial to include in these models the hypodermal compartment, as well as a vascular system and patient-specific cells such as fibroblasts, keratinocytes, and immune cells.

#### 6.5.2 Skin models for microbiome research

Although bacterial contaminations are one of the major concerns to be faced to prevent pathogenic skin infections, the presence of a symbiotic community composed of bacteria, fungi and viruses is essential in maintaining skin homeostasis. Specifically, such symbiotic community, referred to as skin microbiota, is similar to the gut microbiota in composition and second only for bacterial density (i.e., 10^4–^10^6^ bacteria/cm^2^) (Cundell, 2018). It is mainly composed of resident microbes and only a small percentage of transient microbes that can opportunistically increase in the presence of skin diseases. Moreover, the skin microbiota plays a key role in providing nutrients, inhibiting pathogenic growth and regulating epidermal differentiation (Smythe and Wilkinson, 2023). Therefore, understanding the role and functions of skin microbiota in extremely important to provide insights in skin pathologies and facilitating the development and clinical translation of more effective treatments, particularly the use of bioactive textile materials. However, the microbiota complexity and the dynamic interactions between the host and microbial communities are the main challenging aspects to consider in the development of realistic and reliable microbiota models for research (Smythe and Wilkinson, 2023). Moreover, the use of systems mimicking skin structure and physiology is another key feature to fully recapitulate the microbial ecosystem in living skin.

In this context, 3D skin equivalents represent promising candidates for studying host-microbiome interactions combining well-differentiated tissue layers with key skin microbiome components that closely resemble native skin tissue. For instance, Maloney et al. developed an *in vitro* human skin microbiome model containing both stratified host tissue and a microbial consortium composed of six different bacterial strains (Maloney et al., 2025). Specifically, the model was developed by seeding keratinocytes on transwell inserts, promoting their maturation for 7 days until the achievement of a functional ALI as assessed through Trans-Epithelial Electrical Resistance measurements and lastly, bacteria contaminated. The obtained model provided a stable microbiome for up to several days and was found to be a suitable platform to provide insights in the interactions between host and microbes during the pathogenesis of chronic skin diseases.

A similar approach for *in vitro* skin model design was also explored by van Drongelen and colleagues to study the relationship between filaggrin expression and epidermal colonization by *Staphylococcus aureus (* van Drongelen et al., 2014 *)*. Results demonstrated that reduced filaggrin expression was associated with increased bacteria colonization, a key consequence in epidermal barrier damages ascribed to pathologies such as AD.

Despite 3D *in vitro* skin equivalents are currently considered advanced and reliable platforms for native skin tissue mimicking, the absence of glands, blood vessels, appendages and immune cells could be a limiting issue when microbiota modeling is desired. Indeed, the skin houses microbial communities that inhabit spatially distinct regions based on the cutaneous topography (Costello et al., 2009). For instance, *Cutibacterium* species are mainly found in the pilosebaceous units of sebum-rich areas requiring anaerobic growth conditions (Fitz-Gibbon et al., 2013), while *Staphylococcus* and *Corynebacterium* are colonizers of warm and moist environments such as hair follicles and sweat glands (Smythe and Wilkinson, 2023).

Furthermore, 3D skin models have been further improved by adding such key components of the native skin physiology, namely vasculature and improved immunocompetences. For instance, Abaci and co-workers developed a multi-layer skin equivalent with perfusable and spatially controlled vascular networks by combining the latest microfabrication techniques and primary and induced pluripotent stem cell-derived endothelial cells (Abaci et al., 2016). Results demonstrated the successful engineering of mature epidermis and endothelial barrier, thus paving the way to model exploitation for drug screening. More recently, Kang et al. engineered an even more realistic skin model incorporating hair follicles and epidermal/papillary dermal layers relying on the potentialities of 3D bioprinting (Kang et al., 2022). The obtained constructs were characterized by a microporous network resembling the native skin extracellular matrix, promoted epidermis-dermis interactions mediated by the papillary layer and, spontaneous capability to develop hair pore structures.

#### 6.5.3 3D skin models to pre-clinically evaluate bioactive textiles

Although current 3D skin models are indispensable and intensively used for studying the biological effects of various topically applied compounds on human skin components, their use to evaluate bioactive textiles for dermatological applications remains largely unexplored.

In fact, advancing from traditional 2D to 3D skin models offers a physiologically relevant platform to assess key parameters such as biocompatibility, antimicrobial efficacy, and host–microbe interactions. This transition enhances the predictive power of preclinical studies and strengthens the translational potential of bioactive textiles for personalized clinical approaches. In this line, Bengalli et al., 2021 developed antibacterial textiles aimed at preventing infection by incorporating metal oxide NPs, which are considered as promising antibacterial agents. To evaluate the potential toxicological effects on the skin, the authors evaluated these bioactive textiles using an *in vitro* reconstructed 3D epidermal model, (EpiDerm™), under conditions simulating human sweat (Bengalli et al., 2021a). The translation to 3D skin models allowed the assessment of epidermal permeability to metal ions, the relationship between metal ion release and sweat pH, and ultimately the safety of antibacterial textiles on intact skin.

A similar study evaluated the toxicity of polypyrrole NPs-coated textiles using 3D skin models (Bengalli et al., 2021b). Polypyrrole is a conductive polymer with antibacterial properties, biocompatibility, electrical conductivity, and suitability for application in hydrogels, biosensors and controlled drug delivery textiles. The safety of NPs release from textiles was assessed using the EpiDerm ™ model in combination with Balb/3T3 fibroblasts, through tests evaluating skin irritation and corrosion. The results indicated that polypyrrole particles did not induce toxic effects on skin model, supporting their preclinical safety for skin-contact applications.

Overall, these studies highlight the potential of *in vitro* 3D skin models as sustainable and scalable alternatives for assessing the biological impact of bioactive textiles in human skin components (**Table 1**). Nevertheless, only a limited number of studies have explored the use of 3D skin models as preclinical testing platforms for novel bioactive textiles, probably due to the complexity of the experimental workflow, which involves generating a fully differentiated 3D skin construct, followed by textile contact, and monitoring the model over time. Therefore, greater efforts are needed to advance these applications and expand the knowledge in this emergent area.

**Table 1:** Overview of the experimental models ranging from 1D and 2D systems to advance 3D and animal models used to assess the performance of bioactive textiles in human skin components.

Type of model	Strenghts	Weaknesses
1D model 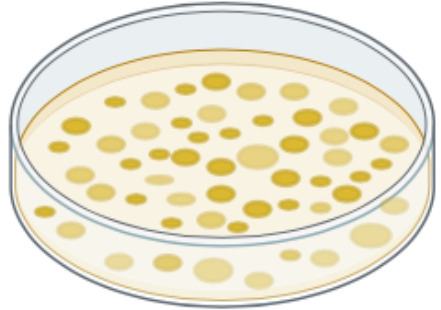 *Simplified system with pathogens in indirect or direct contact with bioactive textiles*	Linear and stepwise conceptual framework to test textile materials through microbiological and mechanistic assays	No inclusion of host cells Providing limited insight into host-pathogen interactions
2D Skin Model 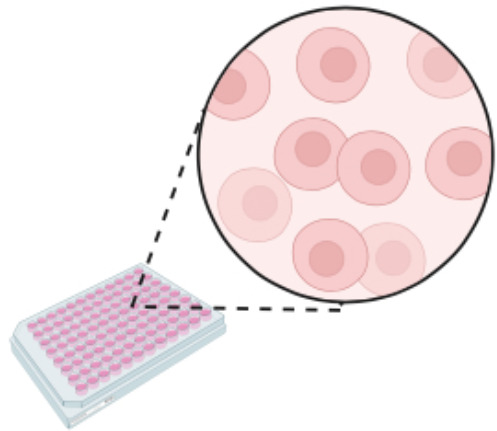 *Monolayers of human skin keratinocytes or human dermal fibroblasts on a flat or rigid plastic substrate, such as a culture dish or microplate*	High cost/effectiveness ratio Standardized and reproducible process Commercially available User-friendly	Inability to replicate the complex *in vivo* skin microenvironment Lack of cell-cell and cell-matrix interactions Limited predictability of drug efficacy
3D Skin model 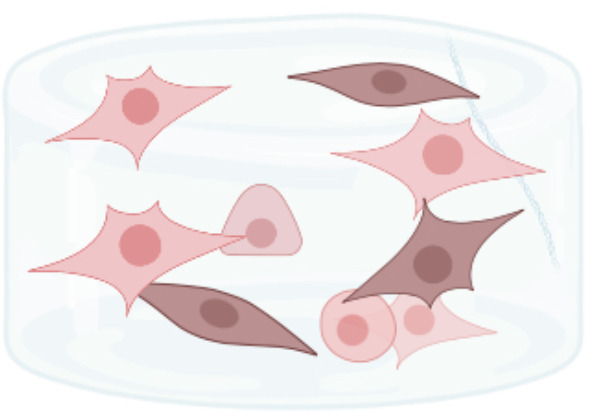 *Co-cultivation of cells (fibroblasts, keratinocytes, and immune cells) in 3D, such as* sp*heroids, organoids, hydrogel-based scaffolds, bioprinted tissues and microfluidics, and organ-on-a-chip*	Capability to reproduce key properties of the human skin, such as architecture and functionality Platform to test not only healthy conditions but also pathological ones, such as persistent inflammation and bacterial contamination Accurate prediction of disease progression and safety and efficacy of innovative biomaterials and therapies Guarantee of cell–cell interactions, tissue-like organization, and physiologically relevant microenvironments	Lack of a standard approach in 3D culture Higher technical expertise, specialized equipment, longer experimental timelines, advanced software, and higher costs required for 3D models
Mouse Skin model 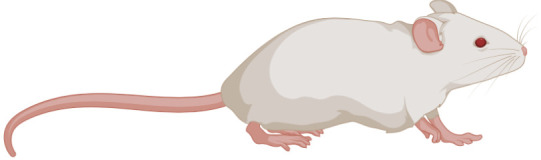 *Animal models mimicking the complex environment of human skin*	Relatively low experimental costs Genetic manipulation to model skin diseases Possibility to promote optical imaging test *in vivo*	Difference in the physiological pathways Difference in bioavailability of pharmaceuticals between humans and mice Different percutaneous absorption of some topical drugs Ethical and regulatory issues related to animal models

## 7 Antimicrobial textiles in the management of skin microbiome dysregulation

The human skin microbiome, composed of a diverse community of commensal microorganisms, plays a vital role in maintaining skin barrier integrity, modulating immune responses, and providing defense against pathogens (Skowron et al., 2021). Disruption of this delicate microbial ecosystem, known as dysbiosis, has been associated with the pathogenesis of various dermatological conditions, including (chronic) wounds, AD, acne vulgaris, psoriasis, hidradenitis suppurativa, and even certain types of skin cancer (Yang et al., 2022). In this dysbiotic state, the balance between beneficial and harmful microorganisms is disturbed, often leading to a reduction in commensal microbial diversity and abundance. This shift creates an ideal environment for the overgrowth of opportunistic or pathogenic species, which can exacerbate inflammation, impair skin barrier function, and contribute to disease progression (Hou et al., 2022).

While conventional treatment strategies for skin microbiota dysregulation usually involves the use of topical or systemic antibiotics, these approaches tend to act broadly and non-selectively, disrupting both pathogenic and beneficial microbial communities (Ito and Amagai, 2022). Such indiscriminate modulation of the skin microbiome may compromise its protective functions and contribute to the emergence and spread of multidrug-resistant bacterial strains, thereby limiting long-term therapeutic efficacy.

As an alternative to conventional antibiotic-based therapies, bioactive textiles with antimicrobial properties are gaining attention as a promising strategy to target pathogenic skin microorganisms (Gauger et al., 2006; Haug et al., 2006; Juenger et al., 2006; Lopes et al., 2015; Suellen Ferro de Oliveira and Kekhasharu Tavaria, 2023; Liu et al., 2024). In fact, their therapeutic potential has been increasingly explored in clinical studies involving patients with skin disorders associated to microbiota dysregulation, where microbial imbalance contributes to disease severity (Gauger et al., 2003; Gauger et al., 2006; Haug et al., 2006; Ricci et al., 2006; Lopes et al., 2015; Schaunig and Kopera, 2017; Thomas et al., 2017; Morand and Hatami, 2019; Ragamin et al., 2021).

A good example is AD, a chronic and relapsing inflammatory skin disorder, associated with skin barrier dysfunction, transepidermal water loss, inflammation, and intense pruritus (Criado et al., 2024). A key factor in AD is the colonization and overgrowth of *Staphylococcus aureus* which exacerbates inflammation and difficult healing (Ogonowska et al., 2020). In this line, several studies have highlighted the therapeutic potential of antimicrobial textiles, namely silver-coated, zinc-coated, chitosan-coated, and cellulose-based fabrics, due to their ability to reduce the proliferation of *Staphylococcus aureus* while alleviating the clinical symptoms associated with AD (Gauger, 2006; Gauger et al., 2006; Jaros et al., 2020; Ragamin et al., 2021).

Another relevant example is acne vulgaris, a multifactorial skin disorder whose pathogenesis involves the overgrowth of *Cutibacterium acnes*, often associated with increased sebum production and localized inflammation. In this context, the use of antimicrobial textiles has shown therapeutic promise. A study investigating a silver-impregnated textile (AEM 5772/5; DermaSilk), known for its antibacterial and antifungal properties, demonstrated a clinically significant reduction in acne lesions on the back, even in the absence of additional treatments or lifestyle modifications (Schaunig and Kopera, 2017).

However, a significant limitation of current clinical studies, mirroring *in vitro* 2D and 3D models evaluations, is the insufficient assessment of how antimicrobial textiles affect commensal members of the skin microbiome. Most investigations have primarily focused on the reduction of pathogenic bacteria, particularly *Staphylococcus aureus*, without evaluating the broader impact on the overall microbial community or the potential role of these materials in restoring or disrupting microbiome balance.

The complex interaction between antimicrobial bioactive textiles, namely those used in clothing, and the human skin microbiome requires deep investigation to elucidate the true implications of such interventions in dermatological care. Understanding how these materials influence microbial diversity and function is essential for evaluating their effectiveness in managing microbiota-associated skin conditions.

**Figure 6:**
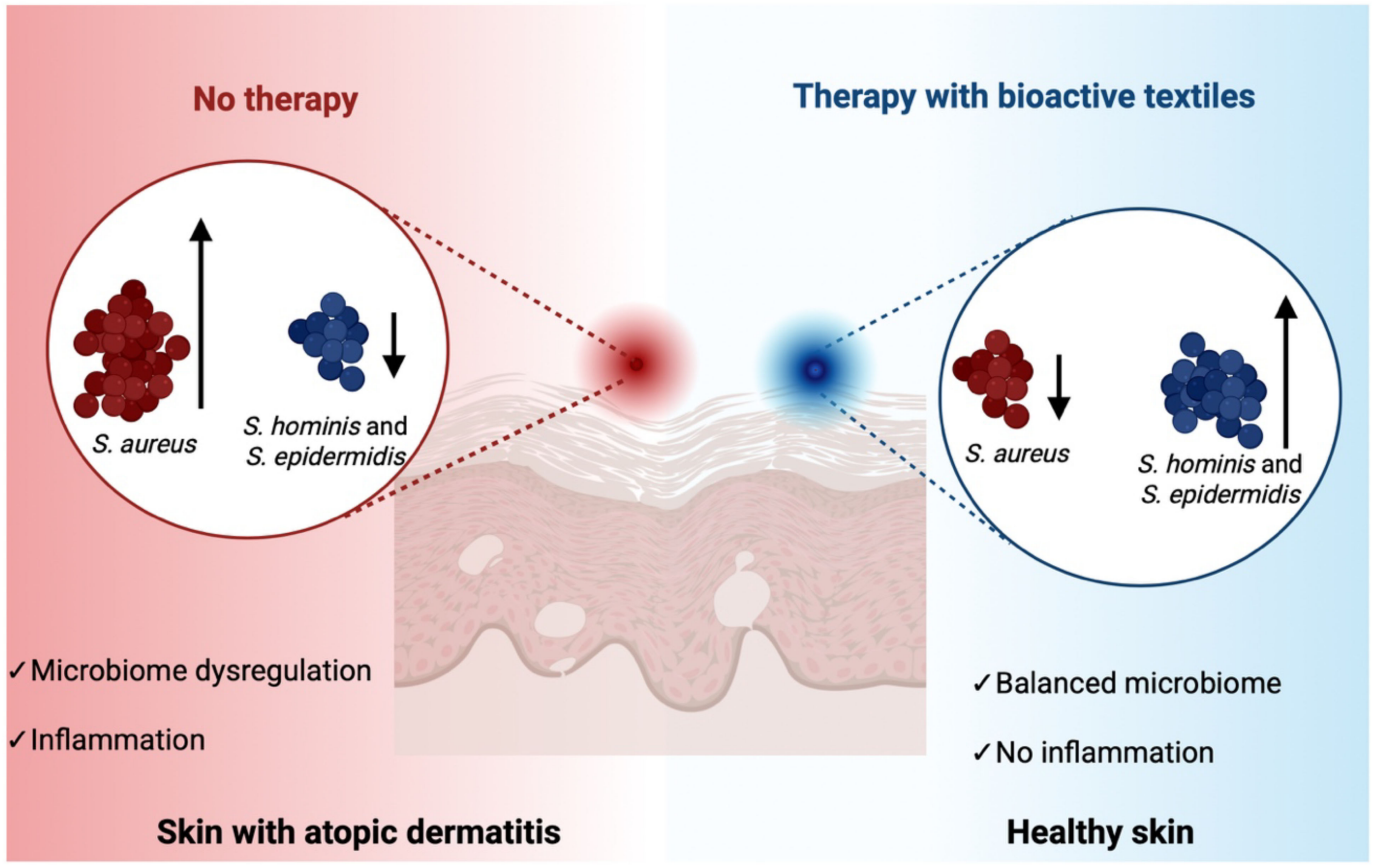
Impact of antimicrobial bioactive textiles on skin microbiome balance in atopic dermatitis (AD). Schematic representation comparing skin microbiome composition in the absence (left) and presence (right) of therapy with bioactive textiles. Without intervention, *S. aureus* predominates over commensal bacteria species such as *S. hominis* and *S. epidermidis*, contributing to microbial dysbiosis and exacerbated skin inflammation. In contrast, therapy using antimicrobial bioactive textiles may selectively suppresses *S. aureus* while preserving beneficial commensal bacteria, promoting microbial balance. The figure was created in Biorender.

Notably, antimicrobial textiles can be engineered to selectively inhibit pathogenic species like *Staphylococcus aureus* while preserving or even promoting the growth of beneficial commensals such as *Staphylococcus epidermidis* and *Staphylococcus hominis*, thereby helping to maintain the skin’s microbial homeostasis (**Figure 6**). Nonetheless, in-depth studies using (advanced) 3D skin models are needed to characterize the full spectrum of microbial responses and determine whether these textiles can reliably modulate dysbiosis in conditions such as AD, acne, or chronic wounds.

### 7.1 Interactions between antimicrobial textiles and the skin immune system

Bioactive textiles with antimicrobial properties interact not only with the skin microbiome but also with the host immune system, either directly or indirectly. The skin serves as both a physical and immunological barrier, where keratinocytes, Langerhans cells, dermal dendritic cells, and resident T cells continuously sense microbial and chemical cues. By modulating microbial populations, bioactive textiles may influence the integrity of this barrier, which is critical for protection against allergens, irritants, and pathogens (Broadhead et al., 2021).

For example, the selective reduction of *Staphylococcus aureus* colonization has been associated with decreased epithelial damage and transepidermal water loss, thereby supporting barrier repair and reducing inflammatory flares in AD. Clinical studies have demonstrated that silver-coated garments reduce *Staphylococcus aureus* burden and improve disease severity in AD patients, highlighting the potential for microbiome-mediated immune modulation (Gauger et al., 2003; Gauger, 2006; Gauger et al., 2006). However, broad-spectrum antimicrobial activity may inadvertently eliminate beneficial commensals such as *Staphylococcus epidermidis*, which play a key role in promoting keratinocyte differentiation, stimulating antimicrobial peptide production, and regulating innate immune responses via TLR2-dependent pathways (Lai et al., 2010). Loss of such protective bacteria may compromise barrier defense and shift immune homeostasis.

Beyond microbial modulation, bioactive textiles incorporating anti-inflammatory or antioxidant compounds (e.g., chitosan, flavonoids, honey-derived products) can directly attenuate innate immune activation by reducing oxidative stress and suppressing pro-inflammatory cytokine release from keratinocytes and macrophages (Lopes et al., 2015; Jaros et al., 2020; de Oliveira and Tavaria, 2024; Freitas et al., 2024). Chitosan-functionalized textiles, for instance, exhibit both antimicrobial and anti-inflammatory properties through the suppression of pro-inflammatory citokynes (Liu et al., 2025). Conversely, uncontrolled release of metal nanoparticles or synthetic antimicrobials may provoke inflammatory responses, undermining tissue homeostasis (Sadrolvaezin et al., 2023).

The role of bioactive textiles in allergic skin responses also warrants attention. Certain textile finishes, including metals, dyes, and preservatives, have been implicated in allergic and irritant contact dermatitis, acting as haptens and triggering immune sensitization in predisposed individuals (Sadrolvaezin et al., 2023). In contrast, textiles engineered with biocompatible or soothing compounds may reduce allergen penetration, alleviate irritation, and mitigate hypersensitivity reactions (Sanchez Armengol et al., 2022). Since allergic inflammation reshapes skin physiology and microbiota composition, explore this crosstalk is essential to understand the complexity of textile–immune interactions.

Taken together, bioactive textiles have the capacity to influence chronic inflammatory responses in conditions such as acne vulgaris, psoriasis, AD, and chronic wounds, where dysbiosis and excessive immune activation are tightly linked. Mechanistic *in vitro* and *ex vivo* work have supported this microbiome–immune cross-talk, while recent advances in immunocompetent, microbiome-inclusive 3D skin models provide translational platforms to study these interactions (Lemoine et al., 2020; Holken et al., 2023). Such models, incorporating keratinocytes, immune cells (e.g., macrophages, T cells), and commensal microbes, allow assessment of whether a given textile formulation reduces pathogenic colonization without inducing sensitization or unintended immune activation. Furthermore, the mouse skin model remains a cornerstone *in vivo* system for investigating host–microbe interactions. Recent work using germ-free murine skin has demonstrated its value in dissecting the distinct contributions of commensals and pathogens to cutaneous immunity, providing critical insights that cannot yet be fully replicated *in vitro* (Uberoi et al., 2025). Such findings are particularly relevant for the evaluation of textile-based interventions, as they allow the evaluation of how bioactive fabrics influence microbial colonization, immune activation, and barrier function within a physiologically relevant context.

In summary, the interplay between antimicrobial textiles, the skin microbiome, and host immunity is complex and highly context-dependent. While some of them show promise in restoring microbial balance and attenuating inflammation, others may have the risk of disrupting commensal ecosystems or provoking adverse immune reactions. Future research should systematically evaluate these outcomes to guide the rational design of safe and effective bioactive textiles for specific dermatological applications.

## 8 Conclusion and future perspectives

Bioactive textiles are emerging as transformative materials in dermatological and healthcare applications, offering specific biological properties and protective functions while aligning with sustainability goals. They represent a shift from passive materials to functional interfaces capable of modulating the skin’s microbiome and barrier function. As demonstrated, bioactive compounds, from metal ions to essential oils and plant-derived polyphenols, have shown promise in reducing skin pathogenic microorganisms’ colonization. However, current evidence remains limited regarding their short and long-term effects on beneficial microorganisms like *Staphylococcus epidermidis*, highlighting the need for a more nuanced understanding of microbiome-targeted textile design.

One of the primary challenges lies in bridging *in vitro* results with *in vivo* outcomes. Conventional 1D and 2D skin models fall in mimicking the dynamic and complex interactions between the skin barrier, immune responses, and microbial communities. Thus, future research must focus on advanced 3D skin models that incorporate not only skin pathogenic members but also commensal microbiota members to better simulate realistic skin-textile interactions. In terms of materials science, the integration of sustainable biopolymers, nanocomposites, and bioactive herbal agents offers a promising avenue for designing eco-friendly and microbiome-compatible textiles (Dasgupta et al., 2025; Yang et al., 2025). Nonetheless, large-scale production, stability of bioactive agents, and regulatory approvals remain hurdles for clinical translation and commercialization.

Future research in bioactive textiles is moving toward a more targeted, sustainable, and technologically advanced approach to skin health management. One of the key priorities is microbiome-specific targeting, wherein textile materials are designed to selectively inhibit pathogenic microbes, such as *Staphylococcus aureus* or *Cutibacterium acnes*, while preserving or even promoting the growth of beneficial skin commensals like *Staphylococcus epidermidis and Staphylococcus hominis*. Achieving this balance is essential for maintaining skin homeostasis and preventing dysbiosis-related conditions. To ensure that these materials are safe and effective, microbiome-integrated testing is becoming an essential aspect of textile evaluation. Conventional antimicrobial testing often fails to account for the complex interactions between skin, fabric, and microbial communities. Therefore, advanced *in vitro* 3D skin models incorporating commensal microbiota, along with metagenomic sequencing tools, are being developed to assess how these textiles influence microbial diversity and composition under realistic conditions.

Another major direction involves the use of sustainable and biodegradable materials. Many bioactive textiles are now incorporating green chemistry principles, using plant-derived compounds, biosynthesized NPs, and biodegradable polymers to reduce ecological impact. This shift not only aligns with environmental sustainability goals but also addresses concerns over toxicity and bioaccumulation associated with synthetic agents.

Lastly, smart textile technologies are gaining momentum. These innovative fabrics can incorporate sensors or responsive release systems that detect skin pH, temperature, or microbial activity, and deliver therapeutic compounds in a controlled way. Such responsive textiles offer real-time adaptability to changing skin conditions, potentially enhancing treatment outcomes and user comfort. In this line, wearable systems are being designed to monitor inflammatory responses or wound conditions and adjust bioactive release accordingly (Broadhead et al., 2021). Together, these future directions highlight a multidisciplinary effort to design textiles that are microbiome-friendly, clinically effective, and environmentally responsible.

In summary, bioactive textiles offer immense promise but require interdisciplinary collaboration and innovation in skin modeling, materials science, and microbiome research to fully realize their clinical and commercial potential.
